# Dickkopf Homolog 3 Induces Stem Cell Differentiation into Smooth Muscle Lineage via ATF6 Signalling*[Fn FN2]

**DOI:** 10.1074/jbc.M115.641415

**Published:** 2015-06-23

**Authors:** Xiaocong Wang, Eirini Karamariti, Russell Simpson, Wen Wang, Qingbo Xu

**Affiliations:** From the ‡ Cardiovascular Division, King's College London BHF Centre, London SE5 9NU, United Kingdom and; the § Institute of Bioengineering, Queen Mary University of London, London E1 4NS, United Kingdom

**Keywords:** cardiovascular disease, cytokine, differentiation, myocardin, smooth muscle, stem cells

## Abstract

Smooth muscle cells (SMCs) are a key component of healthy and tissue engineered vessels and play a crucial role in vascular development and the pathogenic events of vascular remodeling *i.e.* restenosis. However, the cell source from which they can be isolated is limited. Embryonic stem (ES) cells that have the remarkable capability to differentiate into vascular SMCs in response to specific stimuli provide a useful model for studying SMC differentiation. Previous studies suggested that dickkopf homolog 3 (DKK3) has a role in human partially induced pluripotent stem cell to SMC differentiation. Here, we demonstrate that the expression of DKK3 is essential for the expression of SMC markers and myocardin at both the mRNA and protein levels during mouse ES cell differentiation into SMCs (ESC-SMC differentiation). Overexpression of DKK3 leads to further up-regulation of the aforementioned markers. Further investigation indicates that DKK3 added as a cytokine activates activating transcription factor 6 (ATF6), leading to the increased binding of ATF6 on the myocardin promoter and increased its expression. In addition, inhibition of extracellular signal-regulated kinases 1/2 (ERK1/2) promotes the expression of ATF6 and leads to further increase of myocardin transcription. Our findings offer a novel mechanism by which DKK3 regulates ESC-SMC differentiation by activating ATF6 and promoting myocardin expression.

## Teaser

**Background:** The role of DKK3 in vascular SMC differentiation has not been investigated.

**Results:** DKK3 induces myocardin expression via ATF6 signaling.

**Conclusion:** DKK3 is essential for the ESC-SMC differentiation.

**Significance:** This study provides the molecular mechanisms underlying DKK3 regulated ESC-SMC differentiation.

## Introduction

Vascular smooth muscle cells SMCs^4^ are involved in the physiological and pathological processes of the cardiovascular system (1–3). In atherosclerosis, up to 70% of the mass in lesions is estimated to be SMCs or SMC-derived components, *e.g.* extracellular matrix (4, 5). The latest evidence suggests that neointimal SMCs are, at least in part, derived from the differentiation of multipotent stem cells (3). Therefore, understanding the differentiation of stem cells to SMCs can provide new insight of the disease development.

ES cells are characterized by unlimited self-renewal and the potential to differentiate into SMCs *in vitro* (6, 7). They are a useful alternative cell source to obtain SMCs that express specific cell markers such as α-smooth muscle actin (αSMA), smooth muscle 22-α (SM22α), calponin, and smooth muscle myosin heavy chain (SMMHC) (8, 9). Recent work has revealed that DKK3 can regulate human partially induced pluripotent stem cell toward SMC differentiation and is useful for generating tissue-engineered vessels (10). DKK3 is also known as REIC (Reduced Expression in Immortalized Cells), as it was found to be down-regulated in many immortalized cell lines and a number of established human cancer lines (11, 12), functioning as a natural tumor suppressor in human tissues (13). However, little is known about DKK3 functioning during cardiovascular development.

The overall process of SMC differentiation is extremely complex and involves the co-operative interaction of many factors. Myocardin, the transcriptional co-factor of serum response factor (SRF), is found to be required for the expression of many SMC differentiation markers and expressed at higher levels in contractile SMCs than in synthetic SMCs (14). It is crucial in the initial differentiation of SMCs during development (15, 16). Overexpression of myocardin induces ES cells to express multiple SMC genes including α-SMA, SM22α, and SMMHC (17). In atherosclerotic lesions, excess lipids, homocysteinaemia, hypoxic stress and other inflammatory and toxic signals can stimulate endoplasmic reticulum (ER) stress and activate the unfolded protein response in cells (18–20), which activate ATF6 (21). ATF6 is a transmembrane transcription factor that is regulated by ER stress and is involved in adipogenesis and odontoblastic differentiation (22, 23). However, the involvement of ATF6 in SMC differentiation has not yet been found. In the present study, we investigated the role of DKK3 during mouse ESC-SMC differentiation as well as the mechanism for this process.

## Experimental Procedures

### 

#### 

##### Cell Culture and Differentiation

Detailed protocols for mouse ES cell (ES-D3 cell line, CRL-1934; ATCC, Manassas, VA) culture and SMC differentiation were previously described (8, 25). Briefly, ES cells were seeded on gelatin (Sigma) coated flasks and cultured in culture medium (CM), which included Dulbecco's Modified Essential Medium (DMEM) (ATCC), 10% Embryomax Fetal Bovine Serum (FBS) (Millipore), 10 ng/ml Leukemia Inhibitor Factor (LIF) (Millipore), 0.1 mm 2-mercaptoethanol (2-ME) (Life Technologies™), 100 units/ml penicillin, and 100 μg/ml streptomycin (Life Technologies) and 2 mm glutamine (Life Technologies). They were split every other day in a ratio of 1:10. For SMC differentiation, undifferentiated ES cells were seeded on mouse collagen IV (5 μg/ml)-coated flasks or plates in differentiation medium (DM) that contains α-minimal essential medium (aMEM Invitrogen) supplemented with 10% FBS, 0.05 mm 2-ME, 100 units/ml penicillin, 100 μg/ml streptomycin, and 2 mm glutamine. DM was refreshed every day after the second day of differentiation. The cells were cultured in DM for 4–8 days after which they were harvested and further analyzed.

##### Cell Contractility Assays

ES-derived SMCs were washed with PBS, stimulated with 1 mm carbachol or 40 mm KCl (Sigma-Aldrich) in the DM, and monitored under the microscope up to 45 min. Movies of the same field were recorded with time-lapse phase contrast microscopy.

##### Lentiviral Particle Construction and Infection

293-T cells were transfected with the lentiviral vector MISSION® shDKK3 plasmid DNA (Sigma), the packaging plasmids pCMV-dR8.2 (Addgene) and pCMV-VSV-G (Addgene) using Fugene6 (Promega). The supernatant containing the lentivirus was harvested 48 h later and filtered, and the transduction unit (TU) was calculated, as previously described (26). A non-targeting short hairpin ribonucleic acid (shRNA) vector was used to generate the control particles. For the lentiviral infection, mouse ES cells were differentiated for 2 days prior to infection. The cells were then incubated with sh*Dkk3* or non-targeting control (sh*Ctl*) (1 × 10^7^ TU/ml) in DM supplemented with 10 μg/ml of polybrene (Millipore) overnight. Subsequently, fresh DM was added to the cells after which they were incubated for another 2 days before harvesting.

##### Adenoviral Gene Transfer

The mouse *Dkk3* adenovirus was purchased from Abm (Applied Bio Materials) and amplified in HEK 293 cells as instructed by the company. The titration of the adenovirus was assessed at 2.3 × 10^6^ IFU/μl by using the QuickTiter^TM^ Adenovirus Titer ELISA Kit from Cambridge Bioscience (cat. no VPK-110). For the adenovirus infection, cells after 2 days of differentiation were infected with the Ad-*Null* (control particles) or Ad-*Dkk3* adenovirus at an MOI of 345 and harvested 2 days later.

##### Transfection

For the overexpression of ATF6 or silencing of ATF6, cells were seeded on a collagen IV substrate and maintained in DM for 2 days prior to transfection. 0.4 μg of plasmid was mixed with 1.2 μl of FuGENE® 6 transfection regent (Promega) or Lipofectamine® RNAiMAX Transfection Reagent (Life Technologies) per well of a 6-well plate. The solution was then applied to the cells to be transfected. The medium was changed to fresh DM after 6 h. Cells were harvested 2 days after transfection.

##### Conventional PCR (c-PCR) and Quantitative RT-PCR (Q-PCR)

Total RNA was extracted using the RNeasy Mini Kit (Qiagen) according to the manufacturer's protocol. 1 μg of RNA was reverse transcribed into cDNA with random primers by MMLV reverse transcriptase (Promega). 20–50 ng cDNA (relative to RNA amount) was amplified by c-PCR with TaqDNA polymerase (Invitrogen) or Q-PCR with SYBR green (Qiagen) and the appropriate primers.

##### Immunoblotting

Cells were harvested and lysed in lysis buffer (50 mm Tris-Cl, pH 7.5, 150 mm NaCl, and 1 mm EDTA, pH 8.0) supplemented with protease inhibitors and 0.5% Triton by sonication for whole cell lysate. 30–50 μg of protein was separated by SDS-PAGE with 8% Tris-glycine gel and subjected to standard Western blot analysis.

##### Indirect Immunofluorescent Staining

Cells were seeded on gelatin or collagen IV coated slides (Millipore) and cultured in CM or DM. They were fixed with 4% paraformaldehyde for 10 min and then permeabilized with 0.1% Triton X-100 for 10 min at room temperature. 5% serum (same species as the secondary antibody) was used to block nonspecific binding for 1 h at room temperature. The primary antibody was applied and incubated at 4 °C overnight. The samples were then incubated with the appropriate secondary antibodies (Alexa Fluor) for 45 min at 37 °C and counterstained with DAPI (4′, 6-diamidino-2-phenylindole) for 2 min at room temperature. Finally, slides were mounted with fluorescent mounting media (Dako), and images were taken with the fluorescence microscope (Olympus). For every set of experiments, images were captured at the same levels of exposure, gain and offset.

##### Enzyme-linked Immunosorbent Assay

ELISA was performed according to the manufacture's specification (Sino Biolocial Inc.) to measure the level of mouse DKK3 in cell culture supernatants.

##### Co-immunoprecipitation

Cells were infected with the Ad-*Null* or Ad-*Dkk3* adenorius. After infection, the medium was changed to fresh DM with different dilutions of DKK3 antibody (GenScript). The supernatants were harvested, and the complexes were retrieved by resin (Sigma) and precipitated for immunoblotting analysis.

##### Chromatin Immunoprecipitation (ChIP) Assay

ChIP was conducted with the ChIP Assay Kit (Millipore) according to the protocol provided. Briefly, ES cells differentiated for 2 days were infected with Ad-*Null* or Ad-*Dkk3* adenovirus. DKK3 antibody was used in the supernatant after the overexpression of DKK3 to block released DKK3 cytokine. Cells were harvested after cross-linking by formaldehyde. The cross-linked chromatin was sheared by sonication, producing DNA-protein fragments of 300–1000 base pairs (bp) in length. Mouse ATF6 antibody (ChIP grade, Abcam) (IgG as control) coupled to agarose beads was used to immnunoprecipitate the specific complex. The DNA-protein complex was then reversed by digestion of the protein to free the DNA. Finally the purified DNA was used for PCR analysis. The specific primers designed for detecting the binding site of ATF6 are: Forward: GCTGATAACCTCAGGGGCTC; Reverse: CCTTGCCACTCCCATCCATT.

## Results

### 

#### 

##### DKK3 Is Up-regulated during ESC-SMC Differentiation

**FIGURE 1.**
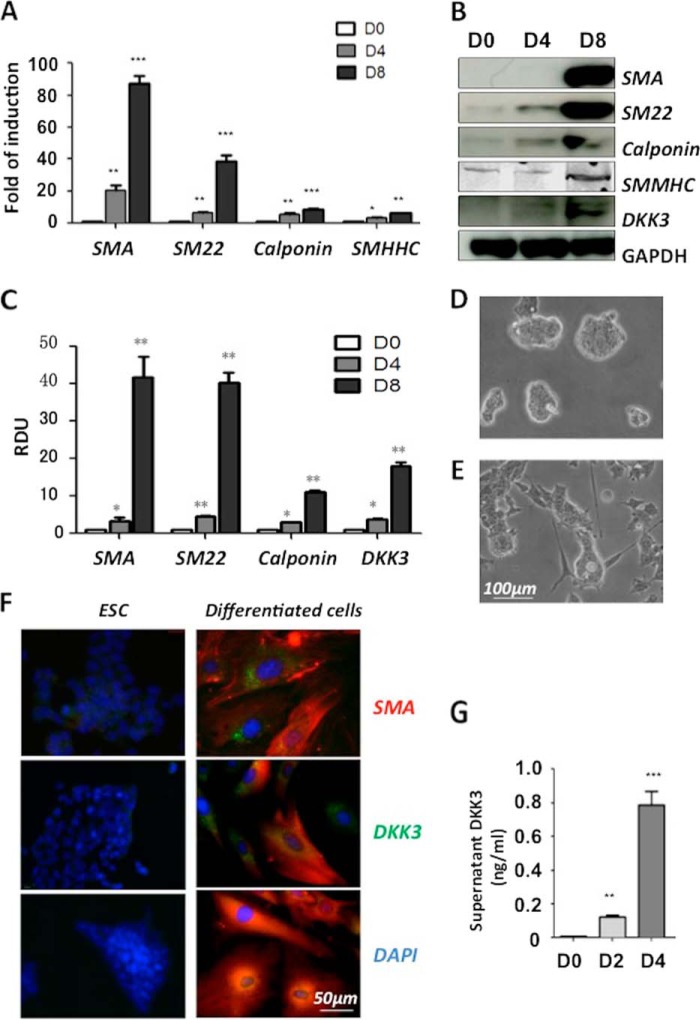
**DKK3 is up-regulated during ESC-SMC differentiation.** For the differentiation, mouse ES cells were seeded on a collagen IV-coated flasks and maintained in DM. Samples were harvested at 4 days and 8 days of differentiation. Undifferentiated ES cells were used as a day 0 control. Q-PCR analysis revealed up-regulation of a full panel of SMC specific markers (*A*) during differentiation. *Gapdh* was used as an internal control. Data shown here as the mean ± S.E. (two-tailed Student's *t* test: *, *p* < 0.05; **, *p* < 0.01; ***, *p* < 0.001). Representative Western blot image (*B*) and quantification of three individual experiments (*C*) showed SMA, SM22, calponin, and DKK3 protein were significantly increased at day 4. RDU, relative densitometry units. Bright field imaging revealed the morphological differences between ES cells (*D*) and 8-day differentiated cells (*E*). Immunofluorescence staining (*F*) revealed that the undifferentiated ES cells expressed low levels of DKK3 protein as well as SMC protein, while the cells seeded on collagen IV-coated plates and cultured in DM for 8 days showed a significant increase of SMC markers and DKK3 protein. Supernatant samples were collected from undifferentiated ES cells and after 2 days and 4 days differentiation. ELISA was performed to measure the concentration of DKK3 protein, and the final results were normalized to every 10^6^ cells (data shown as the mean ± S.E. of three individual experiments) (*G*).

Mouse ES cells were seeded on collage IV-coated flasks and cultured in DM to stimulate SMC differentiation. It was found that SMC markers were successfully induced at both the mRNA (Fig. 1*A*) and the protein (Fig. 1, *B* and *C*) levels at day 4 and day 8, while the expression of DKK3 was also increased during ESC-SMC differentiation (Fig. 1, *B* and *C*). The cells changed from spherical colonies (Fig. 1*D*) to flattened spindle-shaped morphology after 8 days of differentiation (Fig. 1*E*). Importantly, ESC-SMCs displayed the contractility in response to KCl stimulation (Supplemental Movies). Immunofluorescent staining showed a significant increase in the expression of DKK3 and SMC markers after 8 days of differentiation (Fig. 1*F*). Moreover, the detection of secreted DKK3 protein (cytokine) in the cell culture supernatant revealed an increase of DKK3 secretion during the ESC-SMC differentiation (Fig. 1*G*).

##### DKK3 Regulates ESC-SMC Differentiation

**FIGURE 2.**
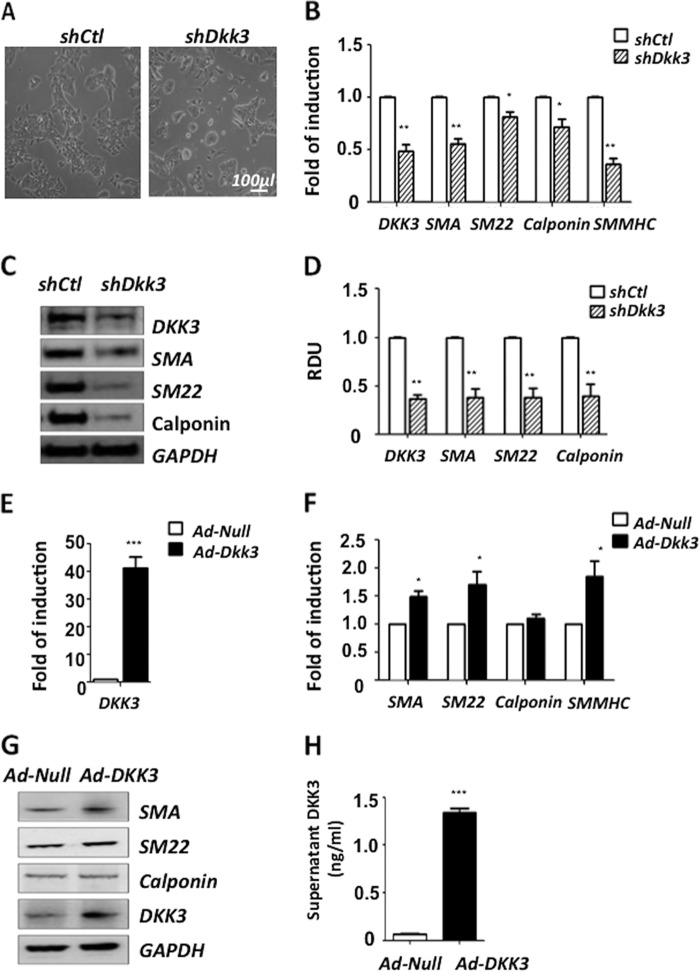
**DKK3 regulates ESC-SMC differentiation.** For the shutdown of DKK3 experiment, ES cells were infected with lentiviruses containing sh*Ctl* or sh*Dkk3* (10^7^ TU/ml complemented with 10 μg/ml polybrene) after 2 days of pre-differentiation. The cells displayed different morphologies (*A*) 2 days post-infection and were subjected to Q-PCR (*B*) and Western blot (*C*) analysis for SMC specific markers. Quantification of Western blot images (mean ± S.E. of three individual experiments) revealed a significant down-regulation of SMC specific markers as well as DKK3 at the protein level (*D*). For the overexpression of DKK3, ES cells were cultured in DM for 2 days before infection with adenovirus particles containing Ad-*Null* or Ad-*Dkk3*. Samples were harvested 2 days after infection. Q-PCR (*E* and *F*) revealed a further induction of SMC markers, such as SM22, SMA, SMMHC after overexpression of DKK3 and Western blot (*G*) showed up-regulation of SMC markers. In addition, ELISA of the supernatant after overexpression of DKK3 revealed a considerable increase in DKK3 cytokine release (*H*). The final results were normalized to every 10^6^ cells (data shown as the mean ± S.E. of three individual experiments).

To uncover whether DKK3 played an important role during the mouse ESC-SMC differentiation process, lentiviral particles containing sh*Dkk3* were introduced to knockdown DKK3 expression in *in vitro* experiments. Cells infected with Sh*Ctl* were used as control group. ES cells were cultured in DM for 2 days before infection. Samples were harvested 2 days after infection. The cells infected with sh*Dkk3* displayed a morphology more similar to ES cells compared with sh*Ctl* (Fig. 2*A*). Q-PCR results demonstrated that knockdown of DKK3 dramatically downregulated the transcription of all the SMC markers (Fig. 2*B*). Western blot analysis also revealed a significant down-regulation of SMC specific markers as well as DKK3 protein (Fig. 2, *C* and *D*). To further investigate the role of DKK3 during ESC-SMC differentiation, ES cells were pre-differentiated for 2 days before adenoviral particles containing Ad-*Dkk3* or Ad-*Null* were introduced. Samples were harvested 2 days after infection. DKK3 (Fig. 2*E*) and SMC markers (Fig. 2, *F* and *G*) were found to be up-regulated after overexpression of DKK3. In addition, the secretion of DKK3 cytokine in the cell culture supernatant was also increased after DKK3 overexpression (Fig. 2*H*).

##### DKK3 Regulates Myocardin Expression

**FIGURE 3.**
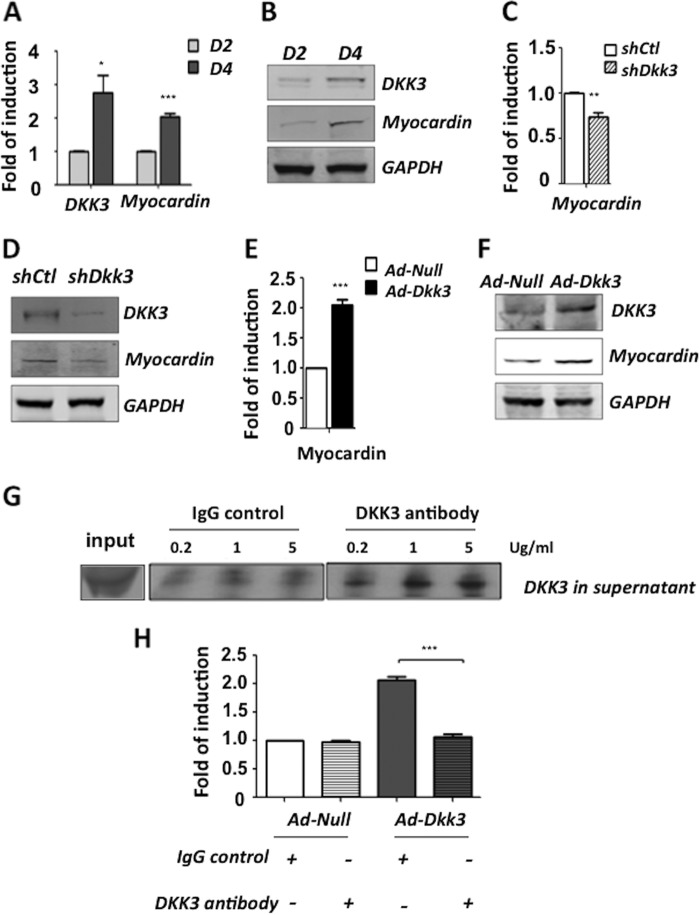
**DKK3 regulates myocardin expression during ESC-SMC differentiation.** During ESC-SMC differentiation, the expression of myocardin was significantly up-regulated from day 2 to day 4 at both the mRNA (*A*) and protein (*B*) levels. Shutdown of DKK3 led to a significant decrease of myocardin expression at mRNA (*C*) and protein (*D*) levels while the overexpression of DKK3 induced further up-regulation of myocardin expression at both the mRNA (*E*) and protein (*F*) levels. To investigate whether the secretion of DKK3 cytokine was important for the DKK3-induced myocardin expression, DKK3 antibody was used to block the effect of DKK3 in supernatant. IgG was used as a control. Addition of DKK3 antibody sufficiently blocked the released cytokine after overexpression of DKK3 (*G*) and Q-PCR analysis (*H*) revealed that myocardin transcription is significantly reduced (mean ± S.E. of three individual experiments, ***, *p* < 0.001) upon blocking the secreted DKK3.

Myocardin is a master regulator of smooth muscle gene expression and was found to be up-regulated in parallel with DKK3 during mouse ESC-SMC differentiation at both the mRNA (Fig. 3*A*) and protein (Fig. 3*B*) levels. In addition, shutdown of DKK3 significantly reduced myocardin expression (Fig. 3, *C* and *D*) while overexpression of DKK3 led to further up-regulation of the myocardin expression (Fig. 3, *E* and *F*). Since DKK3 is a secreted protein, it was of great importance to assess whether DKK3 was exerting its role as a cytokine or as an intracellular component with regards to the regulation of myocardin. To uncover this, a DKK3 antibody was employed to block the effect of extracellular DKK3 upon DKK3 overexpression. ES cells were pre-differentiated for 2 days and then infected with Ad-*Dkk3.* Subsequently, DKK3 antibody or IgG were added in different concentrations (0.2, 1, 5 μg/ml) to the supernatants. It was found that 1 μg/ml DKK3 antibody was sufficient to block the extracellular DKK3 cytokine (Fig. 3*G*) and therefore was chosen as the optimum concentration for further experiments. Cell samples were harvested and assessed by Q-PCR (Fig. 3*H*). It was revealed that blocking of DKK3 cytokine ablated the induction of myocardin transcription upon DKK3 overexpression. These results suggest that DKK3 exerts its function on myocardin expression via its secretion as a cytokine.

##### DKK3 Modulates Myocardin Induction via ATF6 Signaling

**FIGURE 4.**
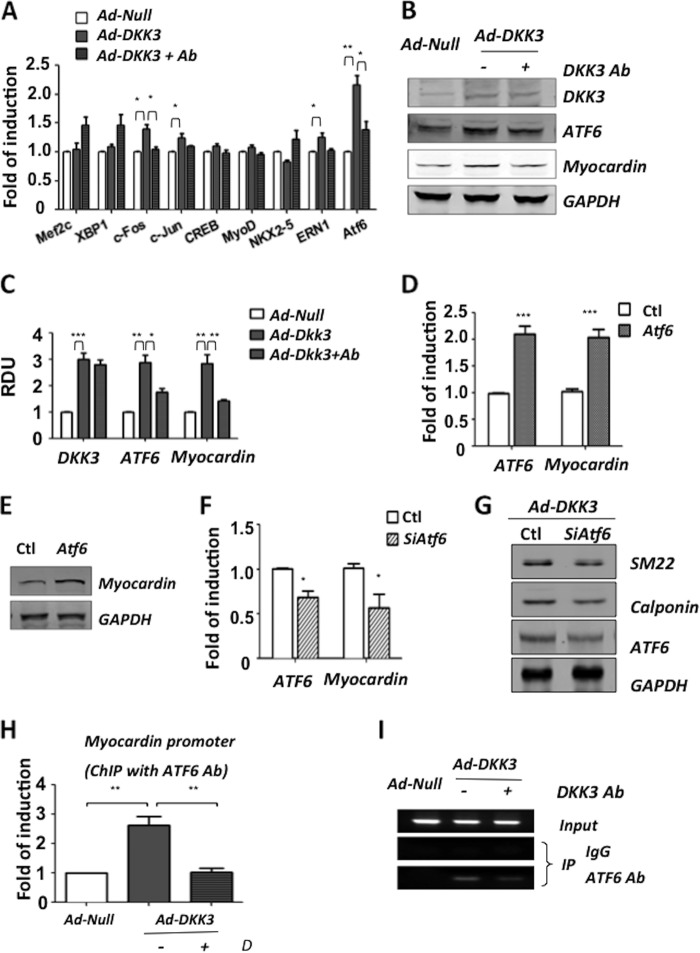
**DKK3 regulates myocardin via transcription factor ATF6.** Treated cells were harvested for PCR analysis indicating the mRNA levels of ATF6 increased upon DKK3 overexpression and reduced upon blocking with 1 μg/ml of DKK3 antibody (*A*). Western blot analysis (*B*) and quantification of three individual experiments (*C*) confirmed the changes of ATF6 at the protein levels. ES cells were pre-differentiated for 2 days and transfected with vectors to overexpress ATF6. The cells were harvested 2 days later, and the result revealed an increase of myocardin expression at both the mRNA (*D*) and protein (*E*) levels. The shutdown of ATF6 by siRNA significantly reduced myocardin transcription (*F*). Shutdown of ATF6 after DKK3 overexpression led to reduced expression of SMC markers (*G*). ChIP assays were performed to confirm the interaction between ATF6 and the MYOCD promoter. Q-PCR (*H*) and c-PCR (*I*) analysis showed that overexpression of DKK3 led to increased binding between ATF6 and the myocardin promoter, an effect which can be diminished after blocking the secreted DKK3.

To clarify the underlying mechanism of DKK3 mediated myocardin induction, the expression of a series of transcription factors with potential binding sites in the upstream promoter region of the myocardin was assessed. 2 days pre-differentiated ES cells were infected with Ad-*Null* or Ad-*Dkk3* and maintained in DM with or without 1 μg/ml of DKK3 antibody. Q-PCR analysis was used to screen the expression of these candidates at the mRNA level (Fig. 4*A*). Finally, ATF6 was selected due to its up-regulation upon DKK3 overexpression and reduced expression as blocking of DKK3 cytokine at both the mRNA (Fig. 4*A*) and the protein (Fig. 4, *B* and *C*) levels. To elucidate whether ATF6 was involved in the regulation of myocardin expression, ATF6 gene was transiently overexpressed by transfection at day 2 of ESC-SMC differentiation or it was silenced at day 2 with siRNA upon the DKK3 induced ESC-SMC differentiation process. It was demonstrated that up-regulation of ATF6 led to further induction of myocardin expression at both the mRNA (Fig. 4*D*) and protein (Fig. 4*E*) levels, whereas the shutdown of ATF6 significantly reduced the transcription of myocardin (Fig. 4*F*). Furthermore, it was shown that shutdown of ATF6 reduced the SMC induction of DKK3 overexpression (Fig. 4*G*). The binding interaction of ATF6 and the myocardin promoter was confirmed by ChIP assay. The results from Q-PCR (Fig. 4*H*) and c-PCR (Fig. 4*I*) analysis demonstrated that there was an increased binding between ATF6 and the myocardin promoter upon DKK3 overexpression. Furthermore, the aforementioned induction was ablated upon blocking of the DKK3 cytokine in the supernatant. These results present novel findings by which DKK3 can induce myocardin expression via ATF6 signaling during ESC-SMC differentiation.

##### ERK Signaling Is Involved in the DKK3 Induced ESC-SMC Differentiation

**FIGURE 5.**
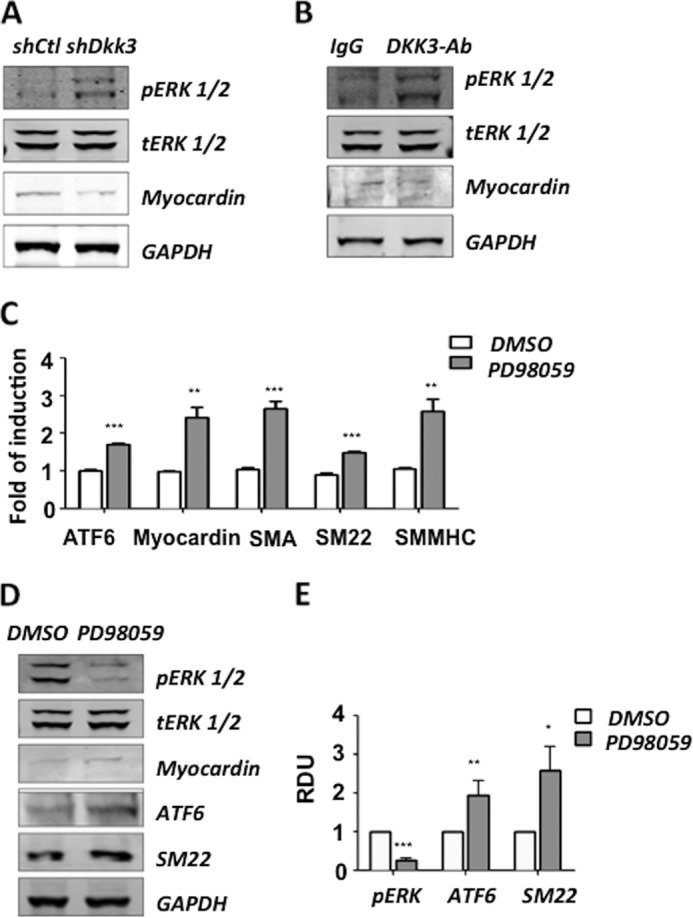
**Inhibition of ERK1/2 promotes DKK3 induced myocardin expression.** The cells were infected with shDKK3, and phosphorylated ERK1/2 was detected by Western blot analysis (*A*). Blocking of the DKK3 cytokine after overexpression of DKK3 rescued the ERK1/2 activity (*B*). To test the role of ERK1/2 in the DKK3 induced myocardin expression, the ERK signaling inhibitor (PD98059) was used after overexpression of DKK3. Cells treated with PD98059 displayed an increase in the transcription of myocardin and SMC markers (*C*). ATF6 as well as SM22 were significantly up-regulated as shown by Western blot analysis (*D*) and quantification of three individual experiments (the mean ± S.E.) (*E*).

It has recently been reported that MEK/ERK signaling plays a role on ATF6 activity in human melanoma cells (27, 28). In this study, we found that shutdown of DKK3 led to activation of ERK1/2 (pERK1/2) (Fig. 5*A*). Moreover, blocking of DKK3 cytokine also showed a regulation of pERK1/2 (Fig. 5*B*). To uncover whether ERK signaling played a role in our system, the MEK/ERK inhibitor PD98059 was utilized. ES cells were pre-differentiated for 2 days and then infected with Ad-*Dkk3.* ERK inhibitor PD98059 or DMSO (vehicle control) was added to cell culture medium after infection. The samples were harvested 2 days later. It was found that ATF6 was up-regulated at both the mRNA (Fig. 5*C*) and protein (Fig. 5, *D* and *E*) levels as ERK1/2 was suppressed. The transcription of myocardin and SMC specific markers was significantly increased upon treatment with PD98059 during DKK3 induced ESC-SMC differentiation (Fig. 5*C*). This suggests that DKK3 may be inhibiting ERK signaling, which leads to the up-regulation of ATF6 myocardin expression.

## Discussion

It has been shown that ES cells can differentiate into SMCs via mechanisms controlled by different signal transduction pathways (8, 9, 29–31). However, the mechanisms that regulate ESC-SMC differentiation are extremely complicated and still not well understood. Although DKK3 has been mainly associated with cancer (11, 32, 33), it is found that recombinant DKK3 protein induces human monocyte differentiation to immature dendritic cells (34). Moreover, recent work in our laboratory has demonstrated that DKK3 plays a crucial role during partially-induced progenitor cell to SMC differentiation by induction of SMC specific markers (10). The aforementioned findings triggered our interest to further investigate the role of DKK3 during the differentiation of SMC from mouse ES cells. In the present study, DKK3 protein is up-regulated in parallel with SMC markers, including αSMA, SM22α, calponin, and SMMHC. DKK3 is essential for ESC-SMC differentiation as knockdown of DKK3 significantly reduced all the SMC specific markers as well as the expression of the SMC master regulator myocardin. Additionally, overexpression of DKK3 leads to a further up-regulation of SMC markers and myocardin.

**FIGURE 6.**
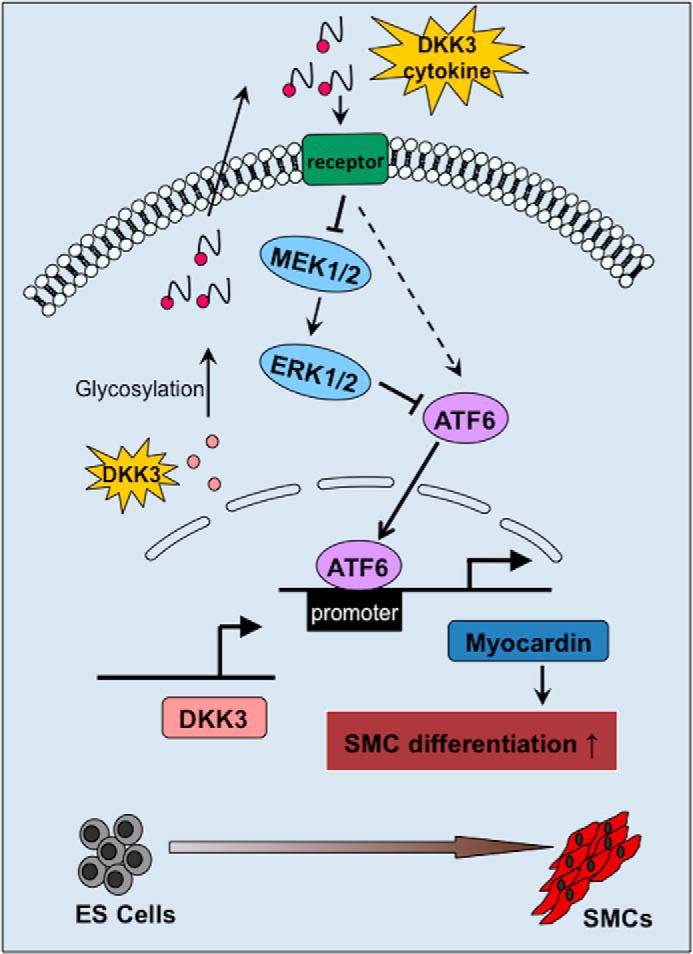
**Schematic representation of the mechanism by which DKK3 regulates ES cells differentiation toward SMCs.** When ES cells were cultured in DM, they can differentiate into SMCs in response to DKK3 stimulation. In this process, DKK3 is up-regulated and secreted into the cell culture medium. In turn, DKK3 cytokine promotes the expression of ATF6 (may via MEK/ERK signaling) and its binding to myocardin promoter, leading to the increased expression of myocardin. The overall effect favorites the SMC gene expression and cell differentiation toward SMCs.

Because of the fact that myocardin regulates SMC marker expression and is important for ESC-SMC differentiation (16, 35), we selected it to be studied further. Furthermore, we found that overexpression of DKK3 highly induces the secretion of DKK3 cytokine that ultimately leads to myocardin gene transcription and myocardin protein expression. Blocking of DKK3 cytokine in the culture medium ablated the aforementioned effect, suggesting that DKK3 can promote *de novo* transcription of myocardin through its role as a cytokine. While trying to understand how DKK3 was transcriptionally regulating myocardin, a number of transcription factors that have the potential to bind on the upstream promoter region of the myocardin was investigated. It was found that among these candidates, ATF6 was the transcription factor with significant expression changes upon DKK3 overexpression and cytokine blocking. ATF6, a key regulator of the ER stress, is known to regulate ER stress-mediated apoptosis during chondrocyte differentiation (36). However, the role of ATF6 in SMC differentiation has not yet been investigated. Experiments conducted in this study revealed that the overexpression of ATF6 further induces myocardin expression, while shutdown of ATF6 diminished myocardin transcription during DKK3 induced ESC-SMC differentiation (Fig. 6). The interaction between ATF6 and myocardin promoter was then confirmed by ChIP assay. Taking together, we show here for the first time that ATF6 serves as a transcription factor of myocardin during DKK3 induced ESC-SMC differentiation.

Although the DKK family is known to be a Wnt inhibitor, the relationship between DKK3 and Wnt signaling is still controversial (37, 38). Additional to Wnt signaling, DKK3 has also been reported to regulate the TGF-β/Smad signaling (39) and ERK signaling (40). It is reported that DKK3 acts as a tumor suppressor in human pancreatic cancer cells via the phosphorylation of ERK (40). It is also suggested that ERK regulates proliferation and migration in rat SMCs (41). In addition, oncogenic activation of MEK/ERK signaling contributes to sustain the activation of IRE1α and ATF6, which is important for melanoma cell survival and promotes the pathogenesis of melanoma (27, 28). In this study, we explored the involvement of the ERK pathway in SMC differentiation and its regulation of ATF6, which has not yet been fully investigated. We found that pERK1/2 is significantly up-regulated upon DKK3 shutdown or when the DKK3 cytokine is blocked after DKK3 overexpression. Inhibition of MEK/ERK by its inhibitor PD98059 significantly increases ATF6 expression at both the mRNA and protein levels and promotes myocardin transcription. The above findings suggest a novel mechanism that DKK3 induces the expression of myocardin via ATF6 signaling, which could be mediated by inhibiting ERK pathways. Thus, targeting DKK3 might provide a new therapeutic strategy for intervention in vascular diseases via regulation of SMC differentiation.

## Author Contributions

Author contributions: Q. X. and W. W. designed research; X. W., E. K., and R. S. performed research; X. W., E. K., Q. X. analyzed data; and X. W. E. K., W. W., and Q. X. wrote the manuscript.

## Supplementary Material

Supplemental Data

## Footnotes

4 The abbreviations used are:

SMC: smooth muscle cell
ATF6: activating transcription factor 6
ChIP: chromatin immunoprecipitation
c-PCR: conventional PCR
DKK3: dockkopf homolog 3
DM: differentiation medium
ER: endoplasmic reticulum
ERK: extracellular signal-regulated kinases
ES cell: embryonic stem cell
ESC-SMC differentiation: ES cells differentiation towards SMCs
Q-PCR: quantitative PCR
RDU: relative densitometry units.
