# Supplementary material for: Dickkopf Homolog 3 Induces Stem Cell Differentiation into Smooth Muscle Lineage via ATF6 Signalling
Source: J Biol Chem. 2015 Jun 23;290(32):19844–52. doi: 10.1074/jbc.M115.641415 (PMC4528144; doi:10.1074/jbc.M115.641415)
Supplement: Supplemental Data [file supp_290_32_19844__index.html]

Dickkopf Homolog 3 Induces Stem Cell Differentiation into Smooth Muscle Lineage via ATF6 Signalling — Dickkopf Homolog 3 Induces Stem Cell Differentiation into Smooth Muscle Lineage via ATF6 Signalling — DKK3 and SMC Differentiation — Supplemental Data 

# Dickkopf Homolog 3 Induces Stem Cell Differentiation into Smooth Muscle Lineage via ATF6 Signalling

## Supplemental Data

- XC Wang \_ JBC MS Sub.pdf (.avi, 1.2 MB) - Supplemental data (Movie 1).
- XC Wang \_ JBC MS Sub.pdf (.avi, 2.8 MB) - Supplemental data (Movie 2).
